# Reviewer acknowledgement 2014

**DOI:** 10.1186/2049-6958-10-2

**Published:** 2015-02-02

**Authors:** Fernando De Benedetto, Claudio F Donner, Claudio M Sanguinetti

**Affiliations:** SS Annunziata Hospital, Chieti, Italy; Mondo Medico Clinic, Borgomanero, Italy; Quisisana Clinical Center, Rome, Italy

## Abstract

The editors of *Multidisciplinary Respiratory Medicine* would like to thank all of our reviewers who have contributed to the journal in Volume 9 (2014).

**Nicolino Ambrosino**

Italy

**Filippo Andò**

Italy

**Spacone Antonella**

Italy

**Sabina Antoniu**

Romania

**Kalliopi Athanassiadi**

Greece

**Bruno Balbi**

Italy

**Carlo Bellieni**

Italy

**Salvatore Bellofiore**

Italy

**Luca Bianchi**

Italy

**Andrea Bianco**

Italy

**Gian Luca Biscione**

Italy

**Victor Borja-Aburto**

Mexico

**Alberto Braghiroli**

Italy

**Claudio Bruschi**

Italy

**Gaetano Caramori**

Italy

**Stefano Carlone**

Italy

**Lucio Casali**

Italy

**Piero Ceriana**

Italy

**Joan Chen**

United States of America

**Gaetano Cicchitto**

Italy

**Gene Colice**

United States of America

**Lorenzo Corbetta**

Italy

**Isabel Couto**

Portugal

**Julio Croda**

Brazil

**Lance Dalleck**

United States of America

**Thuy-Tien Dam**

United States of America

**Silvestro Ennio D'Anna**

Italy

**Paulo de Tarso de Carvalho**

Brazil

**Annalisa de Silvestri**

Italy

**Jacobus de Waard**

Venezuela

**Antonino Di Stefano**

Italy

**Marieke Duiverman**

Netherlands

**Marcia Faganello Mitsuya**

Brazil

**Ibrahima Faye**

Malaysia

**Giovanni Ferrara**

Sweden

**Serhat Findik**

Turkey

**Francesca Fiorentino**

United Kingdom

**Giovanni Fontana**

Italy

**Silvia Forte**

Italy

**Giorgio Fumagalli**

Italy

**Stefano Gasparini**

Italy

**Michael Goodman**

United States of America

**David Griffith**

United States of America

**Christian Guilleminault**

United States of America

**Stefan Hacker**

Austria

**Vijay Hadda**

India

**Ivo Henkens**

Netherlands

**Nidia Aparecida Hernandes**

Brazil

**Jose Luis Izquierdo**

Spain

**Paul Jones**

United Kingdom

**Esen Kiyan**

Turkey

**John Kolbe**

New Zealand

**Christopher Lambers**

Austria

**Mirco Lusuardi**

Italy

**Hitoshi Maeda**

Japan

**Stefano Marinari**

Italy

**Salvatore Mariotta**

Italy

**Fabio Massera**

Italy

**Stefano G. Nardini**

Italy

**Benoit Nemery**

Belgium

**Margherita Neri**

Italy

**Tevfik Özlü**

Turkey

**Berkant Özpolat**

Turkey

**Giovacchino Pedicelli**

Italy

**Girolamo Pelaia**

Italy

**David Phelps**

United States of America

**Pietro Pirina**

Italy

**Jana Plevkova**

Slovakia

**Francesca Polverino**

United States of America

**Mario Polverino**

Italy

**Salvatore Privitera**

Italy

**Yogesh Saini**

United States of America

**Shoji Sanada**

Japan

**Antonio Sanna**

Italy

**Carlo Santoriello**

Italy

**Cosima Schiavone**

Italy

**Gianfranco Schiraldi**

Italy

**Konrad Schultz**

Germany

**Nicola Scichilone**

Italy

**Ashish Sinha**

United States of America

**Yuanlin Song**

China

**Henri Steyaert**

Belgium

**Britt-Marie Sundblad**

Sweden

**Ravi Thiagarajan**

United States of America

**Palle Toft**

Denmark

**Alessandra Toledo**

Brazil

**Renata Trimer**

Brazil

**Ekong Udoh**

Nigeria

**Yasuhiro Usui**

Japan

**Carlo Vancheri**

Italy

**Carlo Alberto Volta**

Italy

**Tobias Welte**

Germany

**Peter Wijkstra**

Netherlands

**Heinrich Worth**

Germany

**Keramettin Yanik**

Turkey

**Dongfang Yu**

United States of America

**Paolo Zamparelli**

Italy

**Pietro Zanon**

Italy

